# A Multicomponent Program to Improve Self-Concept and Self-Esteem among Intimate Partner Violence Victims: A Study Protocol for a Randomized Controlled Pilot Trial

**DOI:** 10.3390/ijerph18094930

**Published:** 2021-05-06

**Authors:** Gemma Sáez, Carla López-Nuñez, Jorge Carlos-Vivas, Sabina Barrios-Fernández, Jorge Rojo-Ramos, José C. Adsuar, Daniel Collado-Mateo

**Affiliations:** 1 Department of Psychology and Anthropology, University of Extremadura, 06006 Badajoz, Spain; 2 Department of Personality, Assessment and Psychological Treatments, School of Psychology, University of Seville, 41010 Seville, Spain; clnunez@us.es; 3 Health Economy Motricity and Education (HEME), Faculty of Sport Science, University of Extremadura, 10003 Cáceres, Spain; jorgecv@unex.es (J.C.-V.); jadssal@unex.es (J.C.A.); 4 Nursing and Occupational Therapy College, University of Extremadura, 10003 Cáceres, Spain; sabinabarrios@unex.es; 5 Centre for Sport Studies, Rey Juan Carlos University, Fuenlabrada, 28943 Madrid, Spain; daniel.collado@urjc.es

**Keywords:** wilderness therapy, intimate partner violence, multicomponent program

## Abstract

Background: Intimate Partner Violence (IPV) is a major public health problem that affects one-third of women aged 15 around the world. Interventions for IPV victims are essential for women’s self-esteem and self-concept recovery. This project aims to assess the effects of an eight-session multicomponent intervention program based on group psychological therapy and adventure activities in (1) self-esteem, (2) self-concept, (3) body image, (4) self-efficacy and (5) depression symptomatology in IPV victims. Methods/Design: A single-blind, randomized controlled pilot study, with experimental and control group, will be carried out. 34 IPV female victims will be recruited and equally assigned to the experimental (*n* = 17) or the control (*n* = 17) group. Primary outcome measures will include self-esteem, while secondary measures will be focused on self-concept, body image, self-efficacy, and depressive symptoms. Intention to treat and efficacy statistical analyses will be also performed. Discussion: This project will explore the effects of a new multicomponent program which includes cognitive-behavioral therapy sessions and outdoor adventure activities on affective and emotional variables, often affected in IPV victims. In addition, orientations to incorporate the main findings into the community based IPV resources and victims’ services will be provided.

## 1. Introduction

### 1.1. The Challenge of Violence Against Women

Violence against women is a major public health problem and a violation of women’s human rights [1]. According to the United Nations, there are different manifestations of violence against women such as intimate partner violence (IPV), sexual violence, or human trafficking, among others [2]. Specifically, IPV is committed by a current or former intimate partner, being the most common form of violence against women [3], and includes physical violence, sexual violence, stalking, or psychological harm [1,4].

Even though there are differences in the prevalence of violence depending on the region, globally almost one out of three women aged 15 and over have experienced physical and/or sexual IPV in their lives [5]. In Europe, studies performed by the European Union Agency for Fundamental Rights [6] showed that 22% of women over 15 years involved in a romantic relationship have experienced physical and/or sexual violence from their partner. In Spain, 32.4% of women older than 16 have experienced any kind of violence from their current or former partner in their lifetime and focusing on the last year 10.8% of women have experienced any kind of IPV [7]. In Extremadura, the Spanish region where the study will take place, 9% of women experienced any kind of intimate partner violence in the last 12 months [7]. During 2020, the Spanish Government Office against Gender-based Violence in its Statistical Portal (see http://estadisticasviolenciagenero.igualdad.mpr.gob.es (accessed on 22 March 2021)) reported that 45 women have lost their lives at the hands of their partners. Since 1 January 2003, a total of 1083 women have been murdered in Spain (updated 22 March 2021).

### 1.2. Psychological Consequences of IPV

The consequences of IPV on women’s health are multiple and well known [8], death being the most severe consequence. García-Moreno et al. [3] concluded that 38% of murdered women were killed by their intimate partner. Other consequences are health and psychosocial impacts. Dutton et al. [9] described how psychological, biological, neurological, behavioral, and physiological alterations were associated with posttraumatic stress disorder (PTSD) among IPV victims, leading to neuropsychological impairments [10]. Following ICD-11 criteria (International Classification of Diseases-11 [11]), PTSD and Complex PTSD (CPTSD) might appear as a consequence of women exposure to repetitive IPV and they could re-experience in the future such traumatic events in the present, in the form of vivid intrusive memories, flashbacks, or nightmares. The study carried out by Woods [12] showed high prevalence of PTSD symptoms in abused women. In this regard, women who have suffered IPV have more severe health problems compared with women who have not experienced violence from their partners [3]. Emotional consequences in IPV victims include slower healing compared to physical injuries [13], usually presenting reduced self-esteem [14], self-efficacy [13], body image [15,16], and damaged self-concept [17]. IPV female victims usually have higher prevalence of depression [18].

Suffering IPV often affects the way people see themselves. Focusing on the affective dimension, abused women have lower self-esteem compared to non-abused women [19]. In Tariq’s words “the self-esteem of the victim is completely crushed so much so that she lacks the confidence to preserve autonomy” [19] (p. 30) In relation to the cognitive component of self-esteem, studies have shown how IPV affects self-concept [20]. Penado Abilleira and Rodicio-García [20] concluded that suffering IPV affects the affective self-concept which ultimately leads to a damaged physical self-concept. Moreover, analyzing the different dimensions of the self-concept, female psychological victimization leads to lower academic and familiar self-concept [17].

IPV Victims often report poor physical self-concept, which is included in the Battered Women’s Syndrome as a condition within PTSD [21]. Body image is negatively affected by experiencing sexual [22] and physical abuse [16]. Empirical studies have found that IPV victims with violence-related injuries experienced higher body image concerns, which are a predictor of depressive symptoms [16]. IPV also affects women’s emotional appraisal regarding their body image, because psychological violence involves attacks and insults towards the victim’s body image, leading to feelings of shame toward their body [23].

IPV not only negatively affects women’s image, but also women’s belief in their own ability to successfully execute behaviors to reach their desired outcomes, that is, their self-efficacy [24]. Self-efficacy is crucial to explain IPV female victims’ individual differences in their recovery [25,26]. Previous studies have shown that self-efficacy is negatively affected by IPV [13] and lower levels of self-efficacy are associated with more severe symptoms of depression among IPV victims [26]. Lastly, studies in different countries have shown that IPV victims have higher levels of depression compared to non-victims [27,28].

### 1.3. Psychological Interventions for IPV Victims

Given the negative psychological outcomes described above, it is necessary to test the effectiveness of psychological interventions for IPV victims. Interventions have been carried out in shelters, prenatal clinics, or in the community [29]. We might distinguish between individual and group therapy for battered women. Whereas individual therapy, like cognitive trauma therapy for battered women with PTSD (CTT-BW), is helpful to restore self-esteem and reduce depression levels [30], group intervention has shown to be effective and the most common form of treatment [31]. Group interventions are mainly based on psychoeducational/supporting groups, cognitive and feminist approaches. Psychoeducational and supportive groups have revealed improvements in self-esteem [32,33] and depression [34]. Moreover, Santos et al. [31] demonstrated how a cognitive-behavioral orientation group intervention could improve self-esteem and depression levels in IPV women. Through a group intervention using psychoeducation and cognitive restructuring among other techniques, authors described a reduction of social isolation, clinical symptomatology, and an improvement on self-esteem [31]. In Spain, Santandreu-Oliver et al. [35] highlighted the effectiveness of a weekly ten-sessions group intervention focused on self-concept and self-esteem enhancement. These authors [35] developed an intervention addressing self-concept, cognitive distortions, pathological criticism, positive thinking, goal setting and psychological manipulation identification, in which self-esteem was the core component.

Other types of intervention have been suggested from different disciplines. In this regard, the connection with nature may be an alternative and a complementary approach to arrive at psychological benefits among people with different medical and psychological conditions [36]. Concerning IPV victims, the American Psychological Association, in their guidelines for psychological practice with women [37], recommended the use of wilderness therapy with abused women. This type of therapy encompasses individual and group therapy in natural settings and includes outdoor programs in the wilderness with personal growth, therapeutic, rehabilitation, education, and/or leadership development purposes [38]. The definition of wilderness therapy is closely related to, or even synonymous, with other concepts, such as adventure therapy or wilderness adventure therapy, among others [39]. Bowen and Neill [39] in a meta-analysis concluded that wilderness therapy programs were effective in short and long-term behavioral, emotional, and interpersonal outcomes [39]. Scant research has explored the role of wilderness therapy among IPV victims. An exception is the study carried out by McBride and Korell [40], who described a wilderness retreat for abused women. Although authors did not assess the effectiveness of the intervention on self-esteem or self-concept, they explained how wilderness therapy might create the opportunity to foster personal empowerment among IPV victims. Moreover, Reizvikh [41] tested informal nature therapy with victims of interpersonal violence, highlighting the importance of including a new way of trauma treatment among domestic violence victims. Levine [42], working with a group of sexually abused women, concluded that wilderness therapy helped the victims to deal with body image concerns and self-imposed limitations.

### 1.4. Aim

It is well known that cognitive-behavioral psychological multicomponent programs are the gold standard for the treatment of a wide variety of psychological problems [43]. This kind of treatment program, applied both individually and in groups, is characterized by the combination of several techniques (components) which aims to address the multiple factors involved in the origin and maintenance of specific psychosocial problems. Such modalities of treatment have previously shown their efficacy when compared with individual components and represent a traditional therapeutic approach with solid empirical support (e.g., [44,45]). To our best knowledge, no published research has explored the effects of a multicomponent intervention program to treat self-esteem and self-concept in IPV victims across an outdoor program in a natural, real setting. This project aims to analyze the effects of a multicomponent intervention program on self-esteem in IPV victims. Additionally, secondary aims include assessing the impact of the intervention on self-concept, body image, self-efficacy, and depressive symptoms. Taking into account the objectives explained above, we have designed a multicomponent program based on an indivisible combination of psychological and adventure components which are encompassed as a single approach. Based on the findings identified in the scientific literature and reported above, we hypothesize that, compared with the control group, women in the experimental group will enhance their self-concept, self-efficacy, and self-esteem while reducing their depressive symptoms and their body image concerns. 

## 2. Materials and Methods

### 2.1. Study Design

A single-blinded randomized controlled pilot trial will be conducted following the guidelines collected in The Consolidated Standards of Reporting Trials Statement (CONSORT) methodology for randomized controlled trials [46].

### 2.2. Ethics Approval

The Bioethics and Biosafety Committee at the University of Extremadura approved the performance of this trial (approval number: 187/2020). This study has been registered in the ISRCTN Clinical Trials Registry recognized by the World Health Organization (WHO) and the International Committee of Medical Journal Editors (ICMJE) (Trial number: ISRCTN14216182).

### 2.3. Randomization and Blinding

Randomization and blinding procedures will be conducted after inclusion and exclusion criteria have been verified. Participants will be assigned to one of the groups: experimental (multicomponent program) or control. A research team member who will not be directly involved in the trial will create a simple computer-generated randomization sequence using the software Research Randomizer (Version 4.0, Urbaniak G.C. and Plous S., Middletown, CT, USA; http://www.randomizer.org (accessed on 22 June 2013) [47]). The assignment will be hidden with a password-protected file. The researchers involved in the data analysis processes will not be aware of the group to which each woman will be assigned (experimental or control).

### 2.4. Participants

Participants will sign the informed consent form and will meet the following inclusion criteria: (a) women older than 18 years, (b) IPV victims, and (c) living in Extremadura (Spain). Due to the nature of the study (outgoing activities) and because of security reasons, women included in the study are not currently living with their batterer. Additionally, the following exclusion criteria will be applied: reporting (a) physical problems that contraindicate adventure activities; (b) other psychological problems such as diagnosed depressive disorder, serious mental disorder (schizophrenia, bipolar disorder, personality disorder) or eating disorders problems, and (c) not to have Spanish language skills enough to communicate effectively with the study staff. The study information will be distributed through public institutions and private social resources in Extremadura (Spain) including women’s institute and women’s association that might have contact with IPV victims. After asking for collaboration, institutions will publish the study flyers in their social media and notice boards, and psychologists and social workers will send this information directly to IPV victims. Women who voluntary decide to participate in the study will contact directly with the researchers and be informed about the procedure. In addition, we believe that due to the characteristics of the sample, word of mouth could also bring participants to the study. Women in both groups will receive a referral card enlisting IPV community resources, including local and national service telephones.

### 2.5. Sample Size

The sample size was calculated using data from previous studies. First, we considered the mean and standard deviation of the Rosenberg Self-Esteem Scale (Spanish version of Fernández-Montalvo & Echeburúa [48]) used in females who were victims of intimate violence and participated in the study carried out by Santandreu-Oliver et al. [35]. Because there were no similar studies using wilderness therapy to improve self-esteem in IPV victims, the expected improvement was set based on the results from the above-mentioned study [35] where enhancements were slightly higher than 10%, and in the study performed by Santos et al. [31] where the improvements were slightly higher than 20%. Thus, the estimated change was set at 15%. A minimum of 17 participants per group (34 in total) would be needed to detect differences of at least 15% with a power of 80% and an α value of 0.05 [49].

Experimental group: participants will attend a multicomponent program which will include: (1) participation in cognitive-behavioral group-based therapy and (2) outdoor adventure activities in the Valle del Jerte. They will continue participating in their daily activities and usual treatments. The intervention program will be composed of eight sessions that will take place over the weekends. Each session will include both psychological and adventure components. The first part of each session will address the psychological component (e.g., group cohesion and behavioral contract) that will prepare the participants to develop the adventure activity, and the second part will be the adventure sport activity itself (e.g., adventure and multi-adventure pedagogy). All the sessions will be driven and supervised by professionals in the field. During the activities with the psychological component, an IPV specialized psychologist will work on self-concept and will prepare women for the activity to be carried out. The second part of the session will be led by experts in sport sciences with additional ongoing psychological support from the therapist. The sessions comprising the multi-component program are detailed in Table 1.

Control group: participants included in the control group will be encouraged to continue receiving their usual treatment care. Women assigned to the control group will be assessed like those included in the experimental group, taking into account the same psychological and social variables at pre-treatment, post-treatment, and at 1- and 3-months follow-ups.

### 2.6. Measures and Procedures

A variety of instruments will be used to assess feasibility and effectiveness of the multicomponent program (Table 2). The assessment will be conducted at different moments: at baseline, immediately after the intervention program, 1 month after post-intervention assessments, and 3 months after post-intervention.

Sociodemographic information: participants will provide information about relevant sociodemographic variables such as age, gender, educational level, etc. Women will be asked about their main medical records, physical and mental health problems, and IPV victimization using a checklist (whether they have ever suffered psychological or physical violence by an intimate partner, had a protection order because of violence, reported violence to the police, or have benefitted from IPV victims’ psychological or social resources) to determine if they meet the inclusion and exclusion criteria. 

Open-ended interviews: these interviews will be implemented to assess the main experiences of the activities developed in the experimental group. This modality of interview will help researchers to understand both positive and negative participants’ points of view about their experiences during the program, as well as to achieve greater insight into the main topics addressed during such interviews [50]. 

Self-esteem: The Rosenberg Self-Esteem Scale (RSE) [51,52] (Spanish adaptation developed by Vázquez Morejón et al., 2004 [53]), will be used. This self-report scale is the most widely used assessment of global self-esteem. The RSE includes 10-item statements that assess self-respect and self-acceptance feelings. Half of the items are worded positively (items 1, 3, 4, 6 and 7) and the other half, negatively (items 2, 5, 8, 9 and 10). The items are answered on a four-point Likert scale (1 = strongly agree, 2 = agree, 3 = disagree, 4 = strongly disagree), with a scoring range from 10 to 40. This scale is a reliable and valid instrument with high internal consistency (α = 0.72 to 0.88; e.g., [54]). 

Self-concept: The Self-concept form 5 (AF-5 [55]) represents a 30-item questionnaire designed to assess five different self-concept dimensions: academic/professional, social, emotional, family, and physical. Each dimension includes six statements that must be answered according to a dimensional scale ranging from 1 (complete disagreement) to 99 (complete agreement). This instrument is a reliable and valid instrument for the assessment of self-concept in children and adult populations, with high internal consistency in different studies (Cronbach’s alpha ranging from 0.71 to 0.88).

Depression symptomatology: Beck’s Depression Inventory-Second Edition (BDI-II; [56], Spanish adaptation developed by Sanz et al. [57]), represents a widely used 21-item questionnaire to assess the severity of depression through different areas such as sadness, loss of pleasure, suicidal ideation, etc. Scores range from 0 to 63, including different cut-off points to classify patients according to the following subgroups: normal to minimal depression (0 to 13), mild depression (20 to 28), moderate depression (20 to 28), and severe depression (scores above 29). This questionnaire has high internal consistency (α = 0.90 to 0.94).

Self-efficacy: The General Self-Efficacy Scale [58] (Spanish adaptation developed by Sanjuán et al. [59]) incudes 10 items aimed to analyze, through a 10-point Likert-type response, feelings of personal competence in handling different stressful situations. Previous studies have already highlighted its high internal consistency with samples from different countries (α = 0.79 to 0.93) (e.g., [59,60,61]).

Body dissatisfaction: The Body Shape Questionnaire (BSQ) [62] (Spanish adaptation developed by Raich et al. [63]) is a 34-item questionnaire which assesses concerns regarding female body image. This instrument includes four subscales (body dissatisfaction, fear of gaining weight, appearance-related low esteem, and desire to lose weight) measured via a six-point scale, ranging from “never” to “always”. The total score ranges from 34 to 204, and higher scores show higher body dissatisfaction. Previous psychometric studies have demonstrated its high internal consistency (α = 0.95 to 0.97) (e.g., [63]). 

### 2.7. Statistical Analysis

The Shapiro-Wilk test will be conducted to check the normality of data and choose between parametric or non-parametric statistical analyses. Socio-demographic continuous variables will be presented as mean (SD) or median (interquartile range) when appropriate, while categorical variables will be reported as proportions. Between-group differences at baseline will be analyzed using independent samples tests. To evaluate the effects of the program, two types of analysis will be conducted:

1. The effects of the intervention will be evaluated using a repeated-measures ANCOVA test, adjusted by age and baseline values. Cohen’s d effect size is 95% confidence interval and statistical significance for time and group interaction effects is group × time. 

2. The intention to treat analysis will be carried out with all participants who will be randomly assigned to either of the two groups. Multiple imputations will be performed to impute data from patients who may not complete a minimum number of sessions. Data imputation will only be used if the reasons for missing data are not related to our exposure/outcome.

All analyses will be conducted using the SPSS statistical package (version 26.0; SPSS, Inc., Chicago, IL, USA). The significance level will be set at 0.05. 

## 3. Discussion

To our knowledge, the current project will be the first trial conducted in Spain which aims to evaluate the effects of a multicomponent program based on wilderness therapy on the self-concept and self-esteem of IPV victims. This is a pioneering project which combines psychological intervention with physical activities in a natural setting, and both interventions have been showed to be effective [35,40]. This program might help women to gain control of their lives and the group format of the multicomponent program will help them to deal with their isolation and to face challenges, skills which could be applied in their daily lives. This single-blinded randomized controlled pilot trial will aim to assess the efficacy of a multicomponent program that will combine psychosocial group intervention and wilderness activities on emotional skills in IPV women. It is also important to note that this multi-component program will focus on challenging adventure activities, rather than the violence women have experienced in their past relationships, avoiding possible female revictimization. 

The psychological intervention suggested in this study protocol is based on the previously reported benefits of psychoeducational and supportive groups on self-esteem and depression [32,33,34]. One remarkable example is the “You’re Not Alone” group, based on a social learning and cognitive behaviors theory perspective, which offered a 14-week therapy group model for IPV female victims that offers therapeutic interventions and support from other IPV victims, showing improvements in self-esteem and depression levels [33]. We expect that the combination of psychological intervention, adventure activities and interaction with nature will mitigate the negative consequences that IPV victimization has for women, including an improvement in their self-concept, self-efficacy, self-esteem and body image, positively impacting on their depression symptomatology.

Regarding limitations, it should be noticed that all the women will be from the autonomous community of Extremadura, so cultural differences could affect the generalization of the results, so further studies will be needed to confirm the results and make them generalizable to women from other regions. Participants in both groups will be encouraged to continue with their usual activities and will be provided with a referral card enlisting IPV community resources, which could affect the results due to the potential heterogeneity of the therapies in which women are usually enrolled. Moreover, this study is based on a short and heterogenous sample design in which we do not intend to distinguish whether women have suffered psychological violence exclusively, or physical violence too. We are aware that our study comprises a small sample size, which could impose a limitation if there were a loss of participants, considering the duration of intervention and the need to travel to perform experimental group activities. Nevertheless, sample size calculations indicated that the sample is enough to achieve a statistical power over 80%. Another limitation is the failure to control for potential comorbidities (e.g., anxiety disorders, PTSD), and this should be addressed on future studies. It should also be noted that, although we have grouped IPV women into a single group, their heterogeneity in terms of type, frequency, severity, and chronicity of the violence is great. Despite these limitations, the current study protocol summarizes the first randomized controlled pilot trial involving psychological and adventure components aimed to enhance the emotional skills of women who were victims of IPV.

## 4. Conclusions

This study will provide empirical evidence about a novel and empowering therapeutic strategy for improving self-esteem and related psychological issues in women who have experienced violence from their partners. If the suggested intervention proves to be effective, findings from this study will have a larger impact on IPV victims in the Spanish community and, more specifically, in the region of Extremadura.

## Figures and Tables

**Table 1:** Proposed activities of multicomponent program intervention.

Session	Adventure Component ^1^	Psychological Component
1	Adventure and multi-adventure pedagogy	Group cohesion and behavioral contract
2	Calm waters crossing and paddle surf	Body image acceptance and one’s exposure own body
3	Rafting	Activation control techniques
4	Rough waters kayaking	Behavioral activation strategies
5	Canyoning	Self-efficacy improvement
6	4 × 4 trail and caving	Identifying our body limits and capacities I
7	Floating	Identifying our body limits and capacities II
8	Half-mountain crossing. Forest bathing	Knowing how to distinguish between objective and subjective risk

^1^ Activities and their order could suffer modifications in case of unfavorable environmental conditions or when external conditions require it.

**Table 2:** Assessment schedule for both experimental and control group.

Measure (Instrument)	Baseline	Post-Intervention	1-Month Follow-Up	3-Month Follow-Up
Sociodemographic data	X			
Experience ^1^ (Open-ended interviews)		X		
Self-esteem (Rosenberg Self-Esteem Scale; RSE)	X	X	X	X
Self-concept (Self-concept form 5; AF-5)	X	X	X	X
Depression symptomatology (Beck’s Depression Inventory-Second Edition; BDI-II)	X	X	X	X
Self-efficacy (General Self-Efficacy Scale)	X	X	X	X
Body dissatisfaction (Body Shape Questionnaire; BSQ)	X	X	X	X

^1^ The open-ended interviews will only be conducted with the experimental group participants to find out about their main experiences with the activities, either positive or negative.

## Data Availability

Not applicable.
